# Tubal pregnancy delivered at term of a live birth: a case report

**DOI:** 10.1093/jscr/rjaf687

**Published:** 2025-09-28

**Authors:** Samuel A Onuh, Fatima E Aliyu, Blessing Ejawemokie, Mustapha Umar, Abdulwahab H M Mohammed, Katharina Weizsacker

**Affiliations:** Obstetrics and Gynecology, Medecins Sans Frontieres, 3JJG+5P9, Kafin Hausa Road, Jahun 720103, Nigeria; Obstetrics and Gynecology, Medecins Sans Frontieres, 3JJG+5P9, Kafin Hausa Road, Jahun 720103, Nigeria; Obstetrics and Gynecology, Medecins Sans Frontieres, 3JJG+5P9, Kafin Hausa Road, Jahun 720103, Nigeria; Obstetrics and Gynecology, Medecins Sans Frontieres, 3JJG+5P9, Kafin Hausa Road, Jahun 720103, Nigeria; Obstetrics and Gynecology, Medecins Sans Frontieres, 26 Olu Agabi Close, Abuja 900001, Nigeria; Obstetrics and Gynecology, Medecins Sans Frontieres, 14-34 Avenue Jean Jaurès, Paris 75019, France

**Keywords:** ectopic, tubal, pregnancy, *HRLS*

## Abstract

An ectopic gestation is the implantation of the conceptus outside the confinement of endometrial cavity. The commonest sites are the tubes, which are 96% of ectopic gestations. Of the ectopic pregnancies, only the abdominal gestation has been known to deliver at advanced gestation. We report a case of a 32-year-old G2P1, who presented with abdominal pain, fetus floating in the abdominal cavity. She had laparotomy with salpingao-opherectomy. The findings of placenta tissue adherent to the right adnexal and omental tissue attached to the placenta. She was discharged on the seventh day. Advanced tubal gestation is rare.

## Introduction

Ectopic gestation occurs when the conceptus implants itself outside the endometrial cavity. It has been associated with maternal morbidity and mortality. The triad of absent menstrual flow, lower abdominal pain, and vaginal bleeding are common. It accounts for ⁓2% of pregnancies. A common site of ectopic gestation is the fallopian tubes [1]. The same proportion is seen in Nigeria [2]. The diagnosis of ectopic gestation can be attained via radiological or surgical diagnosis. This article describes a case of tubal pregnancy at term which is unique and rare of its kind. The literature is rife with tubal ectopic pregnancy, which mostly are second semester gestation. Only one case has been reported of delivery at 34 week which was preterm [3].

## Case presentation

A 32-year-old G2P1 presented with mild lower abdominal pain. She was unsure of her last menstrual period, but an ultrasound done at presentation put her at term. She had no antenatal care visits, her past medical and surgical history were not significant. Her first pregnancy, labour was uneventful of which she had spontaneous vaginal delivery.

On examination, her vital signs (Temp- 36.8°C, PR-123 bpm, BP-140/103 mmHg, RR-18cyc/min, SPO_2_–100%, Hb-9.1 g/dl). Abdominal examination revealed palpable fetal parts, with no signs of peritonitis. There was no vaginal bleeding.

An ultrasound showed a fetus in longitudinal lie (FHR-130) in the peritoneal cavity with an estimated gestational age of 34 weeks +4 days, separately contracted and empty uterus. An informed consent was obtained for laparotomy.

**Figure 1:**
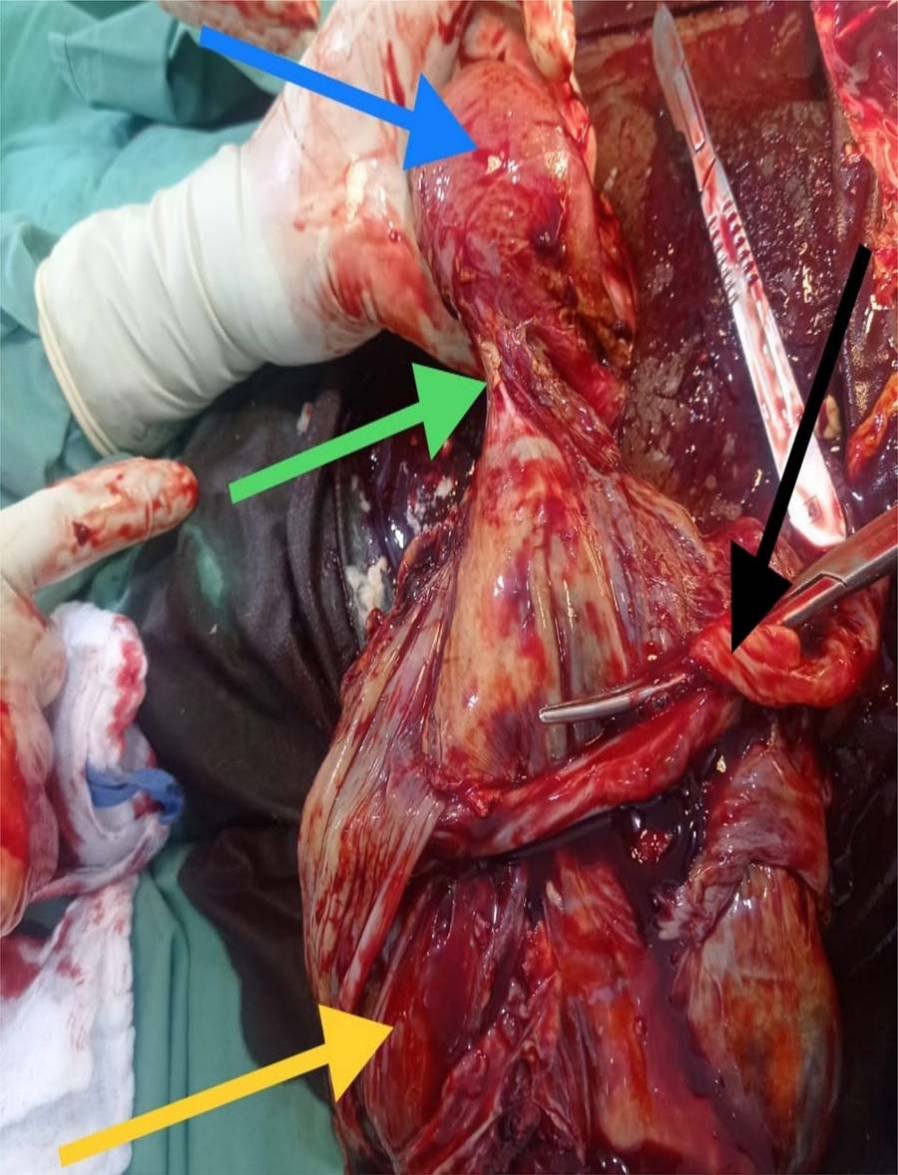
Top left arrow – uterus, middle left arrow – point of attachment of placenta to annexe, bottom left arrow – placenta, middle right arrow – clamped cord.

**Figure 2:**
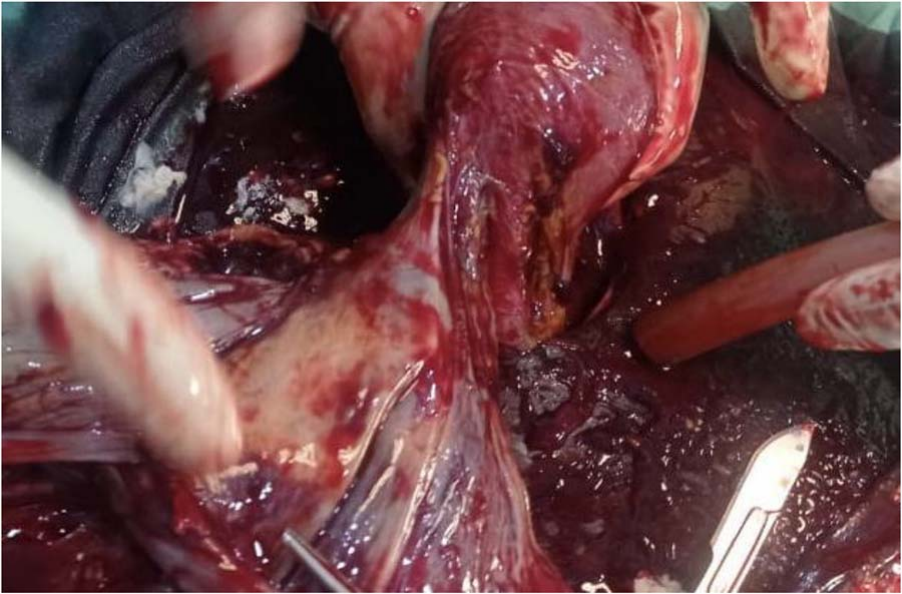
Point of attachment of placenta to adnexae.

**Figure 3:**
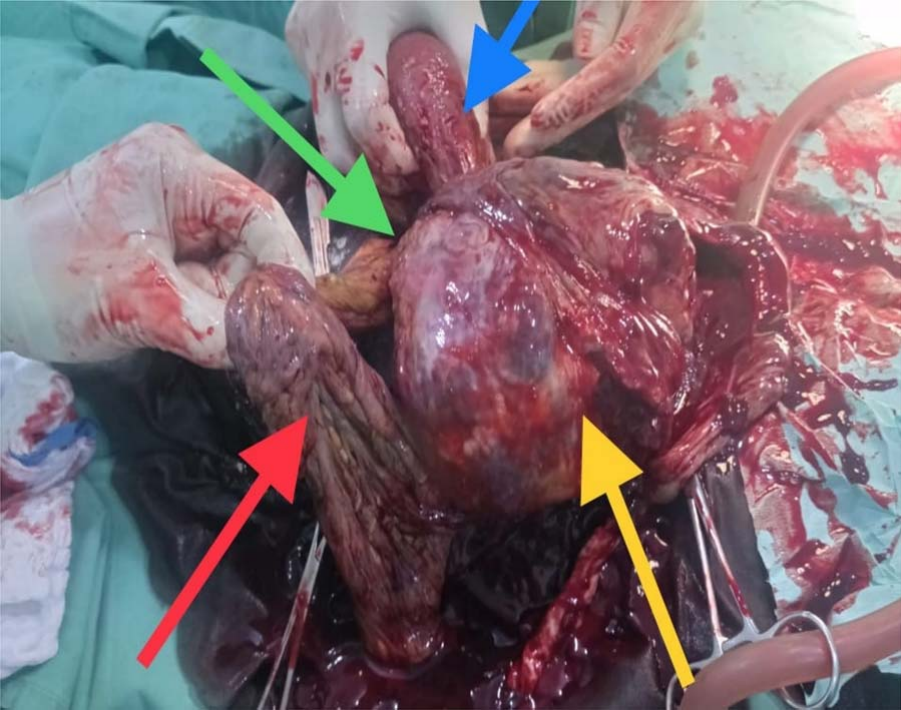
Top left arrow - omental adhesion, bottom left arrow - omentum, top right arrow - uterus, bottom right arrow - placenta.

**Figure 4:**
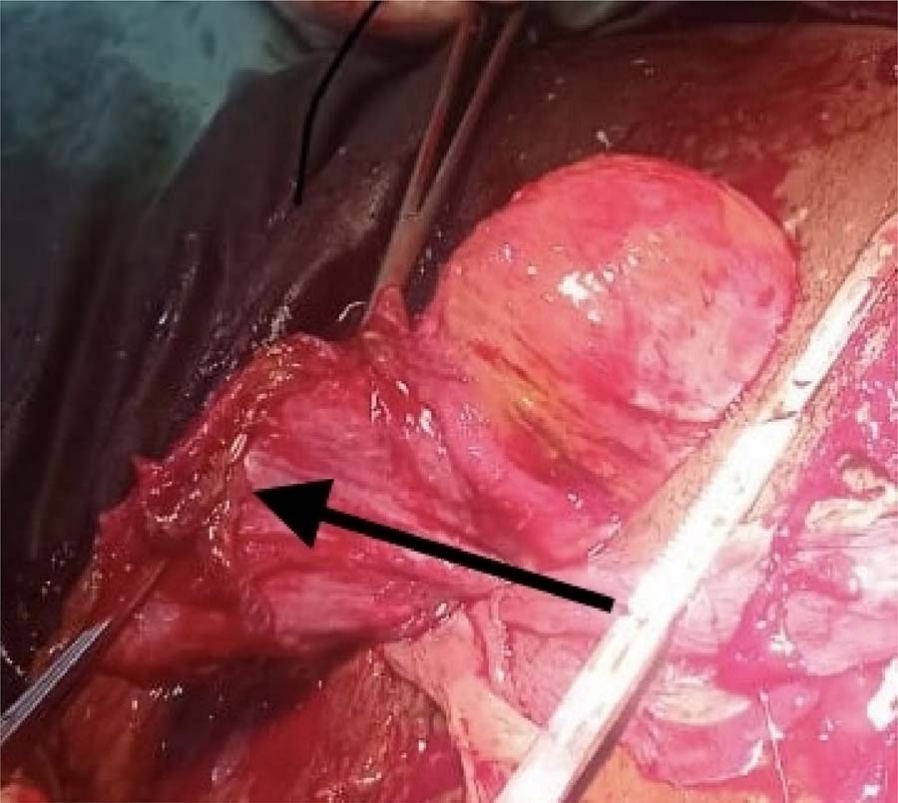
Arrow indicating the clamped and cut part of the adnaxae to which the placenta was attached.

Laparotomy with right salpingo-opherectomy and infra colic omentectomy was performed with the following intra operative findings: a live female neonate with webbed neck that weighed 3.0 kg with an Apgar score of 8,9,10 free in the peritoneal cavity with no amniotic sac and hemo-peritoneum. The placenta was attached to the right ovary, tube and upper part of broad ligament. The distal end of the omentum was attached to the posterior surface of the placental tissue. The uterus, left tube, and ovary were grossly normal (Figs 1–4).

Some clear fluid was noticed in the peritoneal cavity, and an abdominal drain was passed. The rectus sheath and skin were subsequently closed. The patient was transferred to the intensive care unit.

On her first post-operative day, she remained clinically stable with drain output of ˂20 ml of effluent. On the second day, her hemoglobin level dropped from 9.1 to 6.2 g/dl, and her USS showed abdominal collections. A diagnosis of intra-abdominal bleeding was made, and the patient subsequently had a laparotomy with intra operative findings: 2 L of hemo-peritoneum and bleeding from the sutured right adnexal stump. Hemostasis was secured intraoperatively. She had a total six units of blood transfused throughout the course of management. Her abdominal drain was left for 5 days.

**Figure 5:**
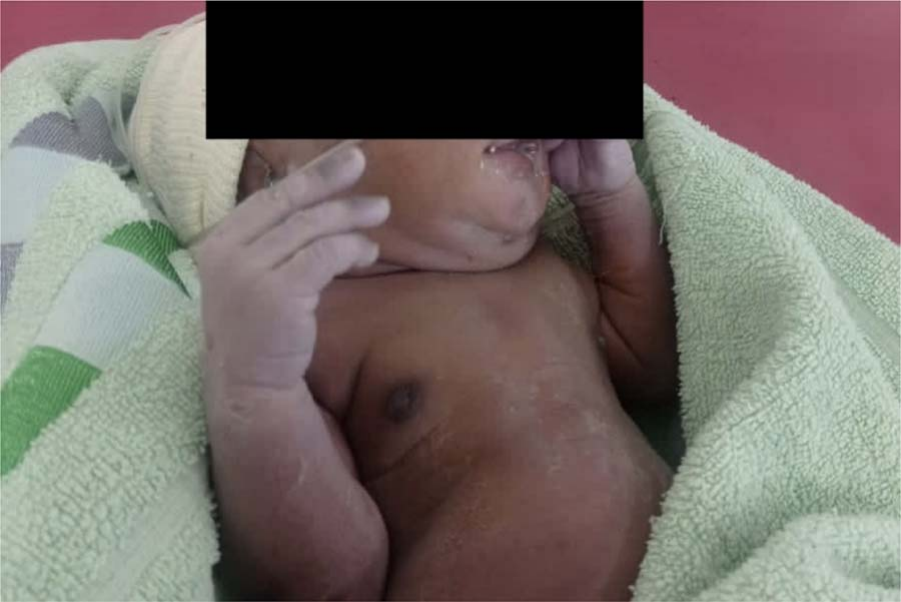
Patient's baby at 21 days of age.

The patient’s vitals remained stable and her abdomen was soft with only mild pain at the surgical site. She was discharged home on the seventh post-operative day with her baby; both were stable. She was subsequently seen at the post-natal clinic with her baby (Fig. 5) on the 14th day after discharge, where her vitals were stable and she had no complaints, with a healed surgical site.

## Discussion

Tubal ectopic gestation accounts for the highest proportion of ectopic gestation [2]. They are usually present within the first trimester, with few cases reported in the second, both in Nigeria and elsewhere [2, 4, 5]. No cases have ever been reported in literature of a term tubal pregnancy. The highest reported was 34 weeks [3]. A study conducted at the Usmanu Dan-Fodiyo University Teaching Hospital in Sokoto, Nigeria, reviewed cases of abdominal pregnancy over a 10-year period. Between January 2000 and December 2010, eight cases were identified among 25 506 total deliveries. The majority of patients were grand multiparas with an average age of 28.1 years [6]. Tubal pregnancy seen after first trimester are thought to survive longer due to the thickened, enlarged tube [7]. Conversely, abdominal pregnancies, though rare, have been found in few reported cases to be carried to term with delivery of a live fetus [8]. This case report presents a rare finding of a live term fetus with the placenta only attached to the adnexa on the right side. This is a tubal pregnancy, since its attachment to the tube and ovaries exclude it from being categorized as abdominal pregnancy.

The viability of this advanced tubal pregnancy may have been aided by the placental vessels receiving a blood supply from the left ovarian artery, hence reducing the migration and attachment of the placenta to other peritoneal tissues (Figs 2 and 4). The patient was managed with laparotomy with an option of leaving the placenta in-situ in case of complex attachment, if removal was not feasible [9]. The index case was managed surgically and removal of the placenta as it was only attached to two structures in the pelvis: omentum and left adnexa (Figs 2 and 3). This was done by clamping and transfixing with suture the adnexal.

Trophoblastic invasion of vascular channels is a complication following ectopic gestation that can cause intra-abdominal bleeding following surgery [10, 11]. As was seen in the index case as the indication for the second laparotomy 48 h after the first surgery, as there were bleeding vessels around the transfixed stump of the adnexa and omental tissue.

The patient remained stable post operatively, with no further complications to mother and baby in clinic follow-up.

## Conclusion

Advanced tubal pregnancy is a rare finding, and this outcome was seen due to the adequate blood supply to the placenta. It could be a diagnostic dilemma as it could be a differential diagnosis of uterine rupture and abdominal pregnancy. Definitive treatment is surgical which involves transfixing of the supply vessel at the attachment of placenta to the tube.

## References

[1] Tonick S, Conageski C. Ectopic pregnancy. ObstetGynecol Clin North Am 2022;49:537–49. 10.1016/j.ogc.2022.02.018 36122984

[2] Olamijulo JA, Okusanya BO, Adenekan MA, et al. Ectopic pregnancy at the Lagos University Teaching Hospital, Lagos, South-western Nigeria: temporal trends, clinical presentation and management outcomes from 2005 to 2014. Niger Postgrad Med J 2020;27:177–83. 10.4103/npmj.npmj_35_20 32687116

[3] Liu Y, Xu X, Liu Q, et al. Advanced tubal pregnancy at 34 weeks with eclampsia and HELLP syndrome: a case report and literature review. BMC Pregnancy Childbirth 2023;23:142. 10.1186/s12884-023-05469-w 36870956 PMC9985250

[4] Drakou A, Cosmo E, Ehrstedt C, et al. Tubal pregnancy with fetus in situ in the 17th gestational week, a case report. Lakartidningen. 2020;117:FX4E. 32430904

[5] Elmoheen A, Salem W, Eltawagny M, et al. The largest tubal pregnancy: 14th week. Case Rep Obstet Gynecol 2020;2020:4728730. 10.1155/2020/4728730 32518701 PMC7260646

[6] Nnadi D, Nwobodo E, Ekele B. Abdominal pregnancy in Usmanu dan-Fodiyo University Teaching Hospital, Sokoto: a 10-year review. J Basic Clin Reprod Sci 2012;1:34. 10.4103/2278-960X.104294

[7] Ngene NC, Lunda O. Ectopic pregnancy in the ampulla of the fallopian tube at 16 gestational weeks: lessons from a case report. Afr Health Sci 2020;20:1895–7. 10.4314/ahs.v20i4.47 34394255 PMC8351844

[8] Gure T, Sultan S, Alishum R, et al. Term abdominal pregnancy with live baby: case report from Hiwot Fana Specialized University Hospital, Eastern Ethiopia. Int Med Case Rep J 2021;14:689–95. 10.2147/IMCRJ.S331195 34616185 PMC8488043

[9] Tegene D, Nesha S, Gizaw B, et al. Laparotomy for advanced abdominal ectopic pregnancy. Case Rep Obstet Gynecol 2022;2022:3177810. 10.1155/2022/3177810 35299756 PMC8923797

[10] Robson D, Lusink V, Campbell N. Persistent omental trophoblastic implantation following salpingostomy, salpingectomy and methotrexate for ectopic pregnancy: a case report. Case Rep Womens Health 2019;21:e00095. 10.1016/j.crwh.2019.e00095 30723694 PMC6350100

[11] Wang T, Li Q. Extratubal secondary trophoblastic implants (ESTI) following laparoscopic bilateral salpingectomy for ectopic pregnancy: problems that have been neglected for a long time. Gynecol Endocrinol 2022;38:608–11. 10.1080/09513590.2022.2078962 35604055

